# Neuroanatomical correlates of language impairment in non-fluent variant of primary progressive aphasia

**DOI:** 10.3389/fnhum.2024.1486809

**Published:** 2024-12-04

**Authors:** Diliara R. Akhmadullina, Rodion N. Konovalov, Yulia A. Shpilyukova, Kseniya V. Nevzorova, Ekaterina Yu. Fedotova, Sergey N. Illarioshkin

**Affiliations:** Research Center of Neurology, Moscow, Russia

**Keywords:** primary progressive aphasia, voxel-based morphometry, functional connectivity, neurolinguistics, apraxia of speech, agrammatisms

## Abstract

**Introduction:**

Non-fluent variant of primary progressive aphasia (nfvPPA) is a neurodegenerative disorder with a predominantly speech and language impairment. Apraxia of speech and expressive agrammatisms along with decreased speech fluency and impaired grammar comprehension are the most typical disorder manifestations but with the course of the disease other language disturbances may also arise. Most studies have investigated these symptoms individually, and there is still no consensus on whether they have similar or different neuroanatomical foundations in nfvPPA. In addition, only few works have focused on the functional connectivity correlates. The aim of our study was to simultaneously investigate functional and structural brain-language associations in one group of nfvPPA.

**Methods:**

Twenty eight patients were enrolled and underwent brain MRI and language assessment. Apraxia of speech, expressive and receptive agrammatisms, repetition, naming and single word comprehension correlates were identified using voxel-based morphometry and resting-state functional MRI (ROI-to-ROI analysis).

**Results and discussion:**

Among the structural correlates, the most common were inferior frontal gyrus (was associated with fluency, both expressive and receptive agrammatisms) and supramarginal gyrus (apraxia of speech, receptive agrammatisms, naming and repetition). Apart from that, neuroanatomical foundations were different for each of the core nfvPPA language domains, including superior parietal lobule involvement in fluency, temporoparietal areas in receptive agrammatisms and supplemental motor area in apraxia of speech. Functional correlations were even more diverse. In general, connectivity decrease between temporoparietal structures was more typical for expressive and receptive agrammatisms, single word comprehension and naming, while apraxia of speech, fluency and repetition showed connectivity disruption mainly among the frontoparietal region and subcortical structures. Overall, extensive structural and functional changes are involved in the development of language and speech disturbances in nfvPPA with distinctive neuroanatomical foundations for each domain.

## 1 Introduction

Non-fluent variant of primary progressive aphasia (nfvPPA) is a neurodegenerative disorder characterized by effortful speech with agrammatisms and articulatory errors and focal atrophy of the left posterior frontal and insular regions. According to the current diagnostic criteria, the presence of at least one of two main symptoms - apraxia of speech (AoS) and agrammatisms - is required for the nfvPPA diagnosis (Gorno-Tempini et al., 2011). AoS occurs due to disruption of complex motor control of speech production and manifests itself in the form of effortful speech with articulatory errors. Grammar impairment in nfvPPA is not limited to the presence of agrammatisms in one’s own speech (expressive agrammatisms) and can also manifest in the form of impaired understanding of complex grammatical structures (receptive agrammatisms) although the former is usually more pronounced. On the contrary, object knowledge, repetition and naming impairment are not characteristic of nfvPPA and are more typical for other primary progressive aphasia (PPA) variants. However, they can manifest with the course of the disease, possibly due to atrophy propagation to other brain areas; when present, they are less pronounced than core symptoms (Botha and Josephs, 2019).

Neuroanatomical correlates of speech and language abnormalities in nfvPPA have long been a subject of interest to researchers mainly due to its focal atrophy, a well-defined and limited speech disturbances and often distinct from vascular areas of brain damage. Previously expressive agrammatisms in nfvPPA were typically associated with lesions in the left inferior frontal gyrus (IFG), precentral gyrus, frontal operculum, middle frontal gyrus (MFG), insula, thalamus, putamen, caudate nucleus, and supramarginal gyrus (SMG) (Tetzloff et al., 2018; Whitwell et al., 2017, 2013; Wilson et al., 2010a, 2010b; Rogalski et al., 2011; Lorca-Puls et al., 2024; Amici et al., 2007b). Although receptive agrammatisms have been studied to a lesser extent, some studies showed that their appearance in nfvPPA can be caused by atrophy of the left IFG (Peelle et al., 2008; Amici et al., 2007a) or the left lateral temporal lobe (Charles et al., 2014; Lorca-Puls et al., 2024). Research on AoS has mostly been focused on primary progressive apraxia of speech (PPAOS) where it is the only clinical presentation. There is still no consensus whether PPAOS is a distinct entity or part of a continuum of nfvPPA (Illán-Gala et al., 2024; Duffy et al., 2021). Nevertheless, studies outline similar bases for the AoS development in these two conditions with involvement of the left premotor cortex, putamen and/or insula in nfvPPA (Whitwell et al., 2013; Lorca-Puls et al., 2024; Ogar et al., 2007) and predominantly the left supplementary motor area (SMA) and premotor cortex in PPAOS (Duffy et al., 2021). Functional studies show that SMA connectivity with other language network regions correlates with AoS severity, further emphasizing the role of this region (Valls Carbo et al., 2022). Several studies of speech fluency showed that it is associated with an atrophy of the MFG, including the premotor cortex, IFG, insula and cingulate cortex of the left hemisphere (Grossman et al., 2013; Rogalski et al., 2011). At the same time, several studies have shown that agrammatisms and decreased speech fluency are associated with different brain regions and possibly have a different neuroanatomical basis in the disease (Mesulam, 2016).

Despite the large amount of works in this area, the main focus of the research was on structural brain changes with fewer studies investigating functional impairment, besides some results were obtained on general group of PPA or neurodegenerative diseases without studying the nfvPPA group separately (Rogalski et al., 2011; Amici et al., 2007a, 2007b). Only few studies have previously assessed all major language manifestations of nfvPPA simultaneously in a single cohort or examined structural and functional correlates concurrently. In addition, there is limited evidence on neural correlates of less common symptoms, such as repetition, naming, and semantic knowledge impairments in nfvPPA. Despite the fact that the current main theory is that these symptoms in nfvPPA have a similar neuroanatomical basis to other PPA variants and appear due to damage to the same regions, this issue remains poorly understood with a small number of studies in this area (Leyton et al., 2019; Premi et al., 2022). The aim of our study was to reveal the structural and functional basis of language impairments in one group of nfvPPA. It should be noted that an additional interest of our study is the neuroimaging study of speech disorders in nfvPPA in the Russian-speaking population. This will be the first data obtained from such a sample.

## 2 Materials and methods

Inclusion criteria for the study were as follows: (1) age over 18 years, (2) a diagnosis of nfvPPA established in accordance with current diagnostic criteria (Gorno-Tempini et al., 2011), (3) no contradictions to magnetic resonance imaging (MRI), (4) the absence of structural brain changes different from those characteristic of PPA on brain MRI. Local Ethical Committee approval was received, and all participants signed an informed consent to participate in the study.

Speech and language examination was performed using the Progressive Aphasia Severity Scale (PASS) (Sapolsky et al., 2014). This scale is used to rate impairment of ten major language domains: articulation, fluency, syntax and grammar, word retrieval and expression, repetition, auditory comprehension, single word comprehension, reading, writing, and functional communication. Symptoms severity in each domain is assessed from 0 to 3 points, including a score of 0.5 points, where 0 is the absence of symptoms, and 3 is severe impairment. Criteria for every score is described for each domain. In our study, we assessed seven domains: articulation, fluency, syntax and grammar, repetition, word retrieval, auditory comprehension, and single word comprehension. Reading, writing, and functional communication were excluded due to the wide variety of reasons that can lead to impairment. We replaced the assessment of articulation with of AoS in order to exclude dysarthria, which can coexist with AoS in nfvPPA and may have other neuroanatomical correlations. Also, when assessing auditory comprehension, only understanding of complex grammatical structures was taken into account as more specific for nfvPPA.

All patients underwent brain MRI on Magnetom Prisma and Magnetom Verio 3 T scanners. The time difference between language assessment and neuroimaging was less than 14 days in all cases. Correlations between symptoms severity and gray matter (GM) atrophy was assessed using voxel-based morphometry (VBM). T1-weighted multiplanar reconstruction sequence was performed to acquire whole brain images, the imaging parameters were: TR = 2,300 ms, TE = 2.98 ms, flip angle = 9°, 1 mm slice thickness, 1 × 1 mm in-plane resolution. All obtained images were quality checked by visual inspection to rule out the presence of artefacts and/or excessive motion. Image preprocessing, statistical analysis and coordinate localization were carried out using the Computational Anatomy Toolbox (CAT12, Jena University Hospital; Germany) in SPM12 (Institute of Neurology; UK) running on MatlabR2020b (Mathworks; Natick, MA, United States). Acquired images were preprocessed using default parameters. Preprocessing in CAT12 included denoising, internal re-estimation for better processing of low-resolution images, image correction for possible errors, affine registration and subsequent segmentation into gray, white matter and cerebrospinal fluid using AMAP (Adaptive Maximum A Posterior) technology. Next, the resulting images were spatially normalized into MNI space in CAT12 and smoothed with an isotropic Gaussian kernel with a full width at half maximum of 8 mm in SPM12. General linear regression was performed to assess associations between each language domain and the GM volume of brain regions. Analysis was adjusted for sex, age, and total intracranial volume, calculated as the sum of GM, white matter, and cerebrospinal fluid volumes. Only clusters with a volume > 50 voxels were included in the results. Due to the relatively small sample size of the groups, the significance level was set at *p* < 0.001, uncorrected for multiple comparisons. Graphical presentation of the results was carried out in the bspmview.

Functional brain-language correlations were investigated next. Patients underwent resting-state functional MRI (rs-fMRI); T2-weighted echoplanar sequence images were obtained with following parameters: repetition time 2,400 ms, echo time 30 ms, flip angle 90°, 2 × 2 mm in-plane resolution, recording 49 slices at a thickness of 2 mm. Obtained images were visually inspected to check for excessive motion or any other artefacts. Image preprocessing, statistical analysis, and results output were carried out using the CONN extension version 21a (Nieto-Castanon and Whitfield-Gabrieli, 2021) running on MatlabR2020b (Mathworks; USA). Preprocessing included image realignment, slice timing correction, outlier detection, segmentation, image spatial normalization to MNI space and smoothing with a Gaussian kernel of 8 mm FWHM with further denoising from white matter, cerebrospinal fluid and possible motion confounding effects followed by frequency filtering to remove BOLD timeseries below 0.01 Hz or above 0.1 Hz. For each of the studied speech domains, functional connectivity (FC) maps between selected regions were obtained (ROI-to-ROI analysis). ROIs were identified using default CONN atlas. Linear regression was performed to locate connections that correlated with symptoms severity with age and sex included as nuisance covariates. Clusters with a *p* < 0.05 corrected for multiple comparisons (pFDR; false discovery rate) were considered significant; the threshold for including individual voxels in clusters was *p* < 0.05. The following areas of interest of the left hemisphere were selected for analysis: IFG (triangular and opercular parts), precentral gyrus, premotor cortex, SMA, posterior and anterior parts of the superior, middle and inferior temporal gyri, temporal pole, SMG, angular gyrus, insula, putamen, caudate nucleus and thalamus. Regions of interest were selected to cover all major brain regions involved in speech function, including regions which atrophy is specific for each PPA variant.

## 3 Results

Twenty eight patients (10 male, 18 female) diagnosed with nfvPPA were enrolled in the study. The median age at the time of study was 64 [57.75; 67] years, median disease duration was 48 [36;63] months. The median years of education was 14 [13.25; 15] years. The severity of dementia, assessed by the Frontotemporal Dementia Rating Scale (FRS), ranged from very mild (14.3%) to severe (7.1%) with a predominance of mild (46.4%) and moderate (32.1%) cases. Motor neuron disease was observed in 4 patients (14.3%), and signs of parkinsonism (but not as a part of corticobasal syndrome or progressive supranuclear palsy) were noted in 8 cases (28.6%).

The results of language assessment using PASS are presented in Table 1. Among language impairments decreased fluency and the presence of expressive agrammatisms were the most common, as expected, and were observed in all patients in the cohort. Less common were receptive agrammatisms (82.1%) and repetition difficulties (89.3%). In two patients the assessment of AoS was impossible due to severe dysarthria, among 26 patients that were tested AoS was found in 85.7% cases. Naming and single word comprehension difficulties were less frequent (67.9 and 35.7%, respectively).

**Table 1:** The severity of speech and language impairments in patients with nfvPPA assessed by PASS.

	Apraxia of speech	Fluency	Syntax and grammar	Receptive agrammatisms	Single word comprehension	Repetition	Word retrieval
0 (no symptoms), n	4	–	–	5	18	3	9
0.5 (very mild impairment), n	4	5	9	13	7	10	7
1 (mild impairment), n	8	10	12	8	2	10	8
2 (moderate impairment), n	8	10	6	2	1	5	4
3 (severe impairment), n	2	3	1	–	–	–	–
NA, n	2	–	–	–	–	–	–
Occurrence among the group, %	85.7	100	100	82.1	35.7	89.3	67.9

**Figure 1:**
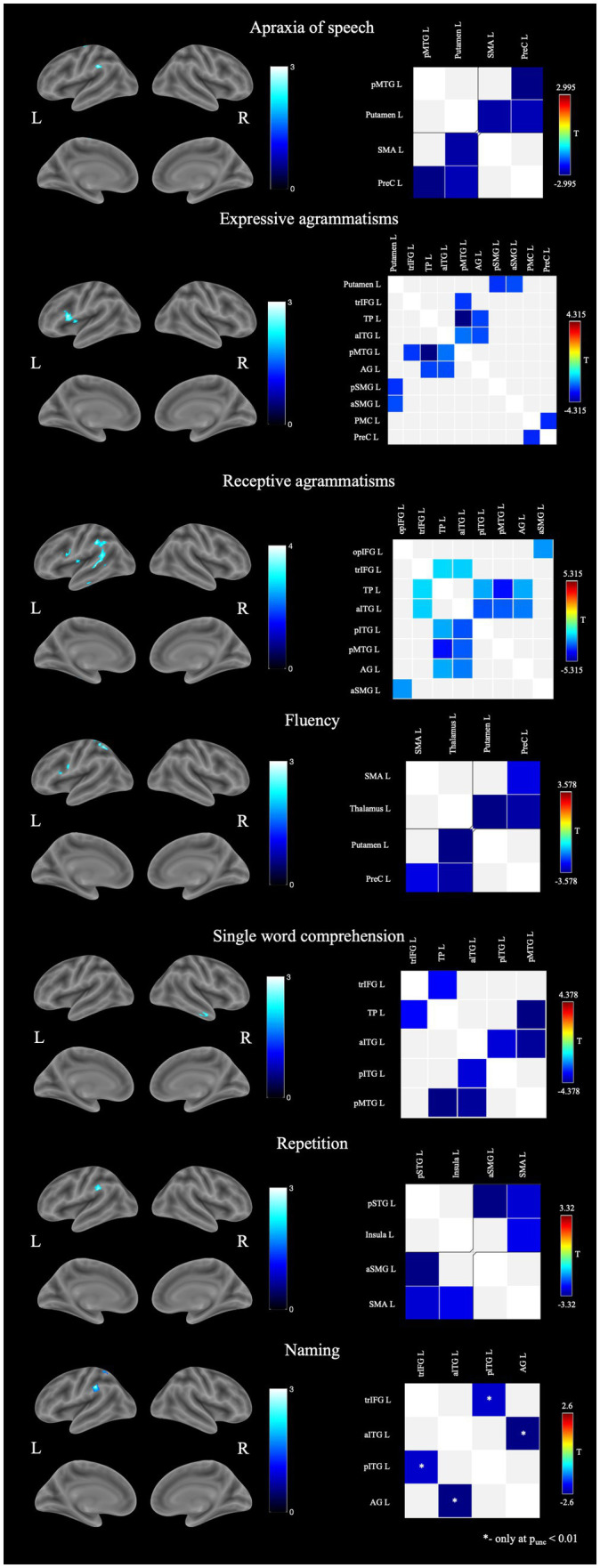
Structural and functional brain-behavior correlates in nfvPPA: on the left—associations with brain gray matter volume, on the right—associations with functional connectivity between brain regions. AG, angular gyrus; aITG, anterior inferior temporal gyrus; aSMG, anterior supramarginal gyrus; L, left; opIFG, inferior frontal gyrus pars opercularis; pITG, posterior inferior temporal gyrus; PMC, premotor cortex; pMTG, posterior middle temporal gyrus; pSMG, posterior supramarginal gyrus; pSTG, posterior superior temporal gyrus; PreC, precentral gyrus; R, right; SMA, supplemental motor area; TP, temporal pole; trIFG, inferior frontal gyrus pars triangularis.

Structural brain-language correlations identified by VBM are presented in Table 2 and in the Figure 1. More “motor” symptoms such as AoS, expressive agrammatisms, and fluency were predominantly associated with the left frontal and parietal lobes volume. Single word comprehension correlated with GM volume of the right anterior temporal lobe, while repetition and naming were localized in the left parietal areas. Brain areas correlated with receptive agrammatisms were the most widespread and included left IFG, insula, inferior parietal lobule and mostly posterior parts of temporal lobe.

**Table 2:** Statistically significant correlations between language impairment and gray matter volume of brain regions in nfvPPA.

Cortical region	Cluster size	*T* value	MNI coordinates
Apraxia of speech			
Supplemental motor area, L	93	3.19	−10, −12, 72
Supramarginal gyrus, L	81	3.03	−58, −33, 34
Expressive agrammatisms			
Inferior frontal gyrus, pars opercularis, L	767	3.62	−54, 8, 22
Insula, L		3.57	−42, 9, 3
Receptive agrammatisms			
Inferior temporal gyrus, L	281	4.91	−45, −26, −27
Inferior parietal lobule, L	1,105	4.58	−58, −46, 40
		4.53	−54, −36, 44
		4.42	−57, −48, 51
Posterior middle temporal gyrus and supramarginal gyrus, L	893	4.51	−58, −39, 3
Inferior frontal gyrus, pars opercularis, L	260	4.08	−45, 3, 18
Insula, L	153	3.99	−38, −10, 14
Fluency			
Inferior frontal gyrus, pars opercularis, L	107	3.29	−56, 10, 26
Superior parietal lobule, L	93	3.23	−28, −45, 58
Precentral gyrus, L	65	3.10	−16, −14, 70
Single word comprehension			
Inferior temporal gyrus, R	62	3.77	64, −9, −27
Repetition			
Supramarginal gyrus, L	50	3.05	−58, −33, 33
Naming			
Superior parietal lobule, L	52	3.22	−26, 42, 69
Supramarginal gyrus, L	74	3.16	−57, −33, 36

L, left hemisphere, R, right hemisphere.

Associations between speech disturbances and functional connectivity (FC) were more widespread (Figure 1). AoS severity correlated with the decreased connectivity of the left frontal lobe regions with the putamen and the posterior middle temporal gyrus (pMTG). Decreased fluency was accompanied by a disruption of connections between the precentral gyrus and the SMA with subcortical regions. Associations of grammar impairments (both in speech production and comprehension), as well as abnormal single word comprehension, were the most extensive and involved connections within the frontal, parietal and temporal regions. Repetition disturbances, in contrast, were associated with FC between only four regions - the SMA, the insula, the posterior superior temporal gyrus (STG), and the SMG. Naming did not show any suprathreshold functional changes with pFDR <0.05. When changing statistical significance to p_uncorrected_ < 0.01 naming was associated with decreased FC between anterior inferior temporal gyrus (aITG) and angular gyrus as well as IFG pars triangularis (trIFG) and posterior inferior temporal gyrus (pITG).

## 4 Discussion

In the course of the study we used VBM and rs-fMRI techniques to obtain data on structural and functional changes that accompany speech and language disorders in nfvPPA. In addition to the core manifestations (AoS, fluency, expressive and receptive agrammatisms), less frequent symptoms such as single-word comprehension, naming, and repetition difficulties that can occur throughout the course of the disease have also been studied. Different patterns of structural and functional changes were identified for each of the language domains.

### 4.1 Apraxia of speech

In our sample, AoS was present in the majority (85.7%) of cases and was more often of mild (33.3%) or moderate (33.3%) severity. According to the VBM data, its severity correlated with atrophy of the SMA and SMG of the left hemisphere - structures responsible for articulation and phonological processing, respectively (Deschamps et al., 2014; Graves et al., 2023; Hertrich et al., 2016; Sliwinska et al., 2012). SMA plays a significant role in the complex planning and initiation of motor acts, including articulation, and its damage is commonly found in AoS, especially in PPAOS (Utianski et al., 2018; Josephs et al., 2012). In addition to the structural lesions, AoS also correlated with FC decline between the SMA and the putamen. These findings are consistent with a number of other studies in nfvPPA: according to Botha et al., FC between the SMA and other structures of the language network correlated with the severity of AoS in PPAOS; Carbo et al. showed that damage to white fibers between the SMA and putamen is associated with AoS, but not agrammatisms; and a study by Mandelli et al. showed that degeneration of the white matter tracts between SMA, the precentral gyrus, and putamen was one of the most significant abnormalities in nfvPPA (Botha et al., 2018; Valls Carbo et al., 2022; Mandelli et al., 2014). The association of AoS with the SMG volume was a less expected finding. It should be noted that the SMG is closely connected with the IFG pars opercularis (opIFG) and the premotor cortex and carries out integration between the phonological and articulatory components of speech (Gow, 2012; Sarubbo et al., 2016). It can be assumed that damage to the SMG may lead to a deterioration in this integration and, as a result, exacerbate articulation errors, especially in the context of existing neurodegeneration. It has also been described that lesions of the SMG can lead to other types of apraxia (Park, 2017). In addition to SMA, AoS in our study was also correlated with decreased connectivity of the precentral gyrus with both the putamen and the pMTG. The putamen has direct connections with the primary motor cortex and also participates in sequence learning and control over motor acts, including articulation (Moustafa et al., 2018; Viñas-Guasch and Wu, 2017). A decrease in this connection can lead to disruption of this regulation and be expressed in apraxia. Decreased FC between the precentral gyrus and the pMTG may serve as a reflection of disintegration between the phonological and motor components, which was also revealed in the structural GM analysis as correlations with the SMG volume. Thus, AoS in our sample was associated with damage to the structures and connections that carry out complex motor control of movements, as well as with disruption of connections between the phonological and articulatory speech components.

### 4.2 Expressive agrammatisms

Along with apraxia of speech, expressive agrammatisms are the second core symptom of nfvPPA. In our sample, expressive agrammatisms were found in all patients, but in contrast to AoS, they were predominantly of minimal (32.1%) or mild (42.9%) severity. According to VBM results, its severity correlated with atrophy of the left opIFG and left insular lobe, which is consistent with the data of previous studies. The opIFG is an essential region for speech and language, and has been repeatedly associated with grammar impairment, including in nfvPPA (Whitwell et al., 2013, 2017; Tetzloff et al., 2018). The insula has extensive connections with the frontal lobe structures, including the IFG; its atrophy is characteristic to nfvPPA and was previously associated with the aphasia severity in the disease (Mandelli et al., 2016; Grossman, 2012).

Functional correlations were more widespread, involving not only frontal lobe but also the parietotemporal regions and the putamen. The identified FC disruption can be divided into several groups: FC decline between the ventral stream components (pMTG, angular gyrus, temporal pole, aITG, trIFG); between the SMG and putamen; between the premotor cortex and precentral gyrus. Damage to the ventral premotor cortex was previously described in connection with agrammatisms in the nfvPPA group and apparently reflected a decrease in speech fluency, which may explain our results (Lorca-Puls et al., 2024). Other studies have previously also shown a connection between the putamen and the SMG in grammatical disturbances in nfvPPA (Whitwell et al., 2017; Rogalski et al., 2011). However, the question remains whether their damage plays a direct role in the development of agrammatisms or affects the deterioration of grammatical function through connections with the opIFG, insula, and other areas of the left frontal lobe. Our data suggest that the putamen and SMG may play their own role in the implementation of the grammatical function of speech. Previously, coactivation of the putamen and the inferior parietal lobule, including the SMG, was shown during speech paradigms execution among healthy volunteers, another study also identified that there is a direct anatomical connection between the putamen and the SMG (Cacciola et al., 2017; Viñas-Guasch and Wu, 2017). It was also found that vascular lesions of the anterior SMG correlated with the severity of agrammatisms even after adjustment for lesion volume and speech fluency, and a small study of healthy volunteers showed the role of the SMG (along with the angular gyrus and posterior parts of the temporal lobe) in speech production when using more complex, but not simple morphosyntactic constructions (Matchin et al., 2020; Schönberger et al., 2014). Thus, decreased FC between the SMG and putamen may have a direct role in grammatical impairments in nfvPPA, but further research in this area is currently required.

### 4.3 Receptive agrammatisms

Along with expressive agrammatisms, difficulties in comprehension of syntactically complex constructions is also widely present in nfvPPA. In our study, receptive agrammatisms were associated with the GM volume of frontal and temporoparietal areas of the left hemisphere, with the predominance of the latter. Early works in this field showed the importance of the posterior frontal regions, especially the IFG, in grammatical processing and that expressive and receptive agrammatisms have the same foundations (Cooke et al., 2002; Kambara et al., 2013; Friederici et al., 2006; Wilson et al., 2010a). Meanwhile, evidence is currently being accumulated on the significant role of the lateral temporal regions in this process, especially their mid-posterior parts (Matchin and Wood, 2020; Zaccarella et al., 2017). Similar data were obtained in recent studies on receptive agrammatisms in nfvPPA, where correlations were found with temporal but not frontal regions (Lorca-Puls et al., 2024; Charles et al., 2014). Our results, in turn, indicate the possible role of both regions, which has also been shown previously (Tyler et al., 2011). Lorca-Puls et al. in a recent study put forward the theory that IFG degeneration in nfvPPA can lead to the mild difficulties in sentence comprehension, whereas moderate and severe impairment develops while atrophy spreads to the left temporal lobe structures (Lorca-Puls et al., 2024). Our results partially fit this theory, but in addition indicate a significant role of the inferior parietal lobule in this process. The latter can play a role in grammatical comprehension processing by providing auditory-verbal working memory (Deschamps et al., 2014; Emch et al., 2019). For example, inferior parietal lobule coactivation with the IFG was noted in response to syntactically ambiguous stimuli, but it did not correlate directly with the complexity of the presented syntactic constructions (Tyler et al., 2011). A similar mechanism can be observed in nfvPPA, when, due to damage to the primary areas responsible for agrammatic sentence comprehension, large resources of verbal working memory are required to complete the task. In addition, as mentioned above, the SMG can also play its own role in providing grammatical functions. Our functional findings were in line with the VBM results. Receptive agrammatisms correlated with the connections between the opIFG and SMG, the anterior temporal lobe with the trIFG, the posterior temporal lobe, and the angular gyrus. The latter changes are similar to the correlations revealed in expressive agrammatisms, and may generally reflect the increased role of ventral stream regions in grammatical processing in nfvPPA in the terms of neurodegeneration.

### 4.4 Fluency

Non-fluent speech is one of the most distinctive features of nfvPPA and can be used in differential diagnosis with other variants of the disease. Decrease in speech fluency in nfvPPA may be due to grammar and articulation impairments, but also may have its own pathogenesis. A number of studies that simultaneously assessed grammatical impairments and fluency in nfvPPA showed that they are associated with adjacent but different brain regions (Rogalski et al., 2011; Mesulam, 2016). Our work has also revealed predominantly different associations between the two symptoms with overlap in the left opIFG. While agrammatism severity correlated with the opIFG and insula volumes, decreased speech fluency was associated with atrophy of the opIFG, precentral gyrus, and superior parietal lobule (SPL). Correlations with frontal lobe regions may be explained by grammatical and articulatory impairments but the association of fluency with the SPL is less clear. Despite the fact that the direct role of this area in speech production has not been shown, the SPL is involved in maintaining attention (especially visual attention) and, according to some studies, has connections with the salience network regions, which is especially prone to neurodegeneration in frontotemporal dementia including PPA (Alahmadi, 2021; Ardila et al., 2018; Koenigs et al., 2009). At the same time, some studies indicate the influence of executive dysfunction and behavioral changes on speech rate, including in nfvPPA (Grossman et al., 2013). Thus, the association between fluency and SPL volume may be explained by the latter’s role in supporting cognitive and behavioral functions, but this requires further study. The rs-fMRI data for fluency also differ significantly from agrammatisms. Impaired speech fluency was correlated with a FC decrease of the precentral gyrus with the SMA and thalamus, as well as the putamen and thalamus thus affecting areas that provide planning and execution of motor speech. Rather similar correlations were found when assessing apraxia of speech, which emphasizes the role of the articulatory component in slowed speech rate in nfvPPA.

### 4.5 Single word comprehension

Impaired word comprehension is the least typical for nfvPPA and, as a rule, is a specific sign of the semantic variant of the disease (svPPA), where it is associated with the temporal pole atrophy (Botha and Josephs, 2019). In accordance with this, the symptom occurred only in a third of patients (35.7%) in our sample with predominance of minimal and mild severity (70 and 20%, respectively). According to the VBM data, single word comprehension correlated with the volume of the anterior temporal lobe of the right hemisphere. Despite the fact that semantic word knowledge is commonly associated with damage to the left temporal pole, some studies indicate the significance of bilateral damage to this area, and cases of predominantly right-sided lesions in svPPA have also been described (Binney et al., 2016; Snowden et al., 2018). Functional rs-MRI confirms the structural findings. Among the components of the left hemisphere, word comprehension impairment correlated with a FC decrease between the left temporal pole and anterior ITG with the left posterior temporal regions and left trIFG (where semantic information from the temporal pole is usually transmitted). Thus, impairments in single words comprehension in nfvPPA has structural and functional changes similar to that in svPPA, and the appearance of this symptom is most likely associated with the atrophy propagation to the temporal lobes during the course of the disease.

### 4.6 Repetition

Repetition impairment is also not typical for the early stages of nfvPPA, and more specific for the logopenic variant of the disease (lvPPA) (Botha and Josephs, 2019). In nfvPPA repetition difficulties can be observed due to the severe apraxia of speech or dysarthria or at later stages of the disease with the development of severe aphasia or behavioral changes. In our cohort, repetition difficulties were observed in the majority of cases (89.3%), more often (80%) with very mild or mild presentation. Cases with moderate repetition impairment were observed only in patients with moderate to severe disease severity (as assessed by FRS) and aphasia duration of more than 3 years. In all cases, repetition difficulties were less pronounced than the key nfvPPA symptoms - apraxia of speech, decreased fluency and agrammatisms.

As in cases with impaired single word comprehension, our main goal in assessing repetition was to identify whether its impairment in nfvPPA has its own pathogenesis or a common basis with lvPPA. VBM showed correlations with the left SMG, which, along with the angular gyrus, is responsible for short-term phonological memory (Henry et al., 2016). Damage to these areas has been associated with the development of repetition impairment and phonological errors in lvPPA (Lukic et al., 2019; Win et al., 2017). Among functional changes, repetition was associated with FC between posterior STG with the SMG and SMA, as well as between SMA and the insula. The first finding suggests disruptions in connectivity between the dorsal stream components, connected by the arcuate fasciculus, and a deterioration in the process of transferring phonological knowledge to the posterior-frontal regions. The arcuate fasciculus connects areas of the dorsal pathway, which ensures the translation of phonological knowledge into expressive speech and its damage leads to repetition impairment and the development of conduction aphasia, which is symptomatically similar to lvPPA (Ivanova et al., 2021; Bernal and Ardila, 2009). In lvPPA, repetition impairments also correlate with the FC between the perisylvian regions and the lateral frontal and temporal regions, which was partially observed in our work (Mandelli et al., 2023). Thus, some of the correlations we identified are similar to those for lvPPA. At the same time, the correlation of repetition with the FC between the SMA and the insula indicates the presence of a motor component in repetition impairment in nfvPPA, possibly due to articulation disorders. Therefore repetition disorders in nfvPPA may be associated both with structural and functional damage to the perisylvian regions and with the involvement of the frontal regions, which suggests the presence of two possible mechanisms of their development, one of which is specific to nfvPPA and the other is similar to lvPPA.

### 4.7 Naming

Although naming difficulties are not characteristic for nfvPPA and more commonly occur in lvPPA or svPPA, their presence does not exclude the nfvPPA diagnosis. In our study anomia was present in more than a half patients (67.9%) with predominantly very mild (36.8%) and mild (42.1%) severity. Moderate anomia were present in four patients, all of them had disease duration >36 months and moderate to severe disease severity. In all patients naming disturbances were less pronounced than AoS and/or agrammatisms.

Structural correlates included SMG and SPL. As it was mentioned earlier, SMG is one of the areas that provide short-term phonological memory. In accordance with this, damage to SMG leads to difficulties in conversion of sound to articulation. In addition to repetition difficulties, this manifests itself in the form of anomia with intermittent speech with word-finding pauses, forgotten words can be remembered with the help of first syllable or multiple choice clue as semantic knowledge remains intact. This profile overlaps with changes found in lvPPA as naming difficulties in this PPA variant are derived mainly from violations of short-term phonological memory due to atrophy of posterior perisylvian regions (Gorno-Tempini et al., 2008). Therefore both repetition and naming in nfvPPA have some similar foundations as in lvPPA. Correlation of naming with SPL volume is more unexpected. In our work SPL volume was also associated with decreased fluency and we proposed that it could be secondary due to cognitive and/or behavioral dysfunction caused by atrophy. Similarly SPL atrophy could affect naming through its role in providing executive functions as there are some works that accentuate connection between executive dysfunction and naming disturbances (Kiss and Csépe, 2024; Higby et al., 2019).

Functional correlates were identified only after lowering statistical significance to *p* < 0.01. Nevertheless, the data obtained are of some interest. In contrast to the VBM findings, the association of naming with FC decrease between aITG with AG, and pITG with trIFG indicates involvement of semantic rather than phonological component of naming. pITG participates in the perception and semantic processing and transfers data from occipital lobe to the anterior parts of the temporal lobe, the main semantic hub (Hope and Price, 2016). aITG is, in turn, associated with the trIFG, and this connection plays a role in the lexical retrieval of semantic knowledge and aids in semantic processing mostly at the word level (Heide Von Der et al., 2013; Kim et al., 2022). aITG is also connected to AG, which, according to various sources, probably participates in both phonological and semantic speech processing, and has extensive connections with the temporal and frontal cortex (Junker et al., 2023; Kim et al., 2022). AG’s role in semantics remains controversial with various theories from direct role in processing semantic information to serving as a hub to accumulate semantic information and integrate it with broader cognitive processes (Zhang et al., 2024; Seghier, 2013, 2023; Graves et al., 2023; Farahibozorg et al., 2022). Regardless of the exact role, decreased connectivity between the aITG and the AG seemingly indicates violations in the semantic processing and/or integration between semantic and phonological components.

Thus, our data suggests that naming difficulties in nfvPPA may have multidirectional foundations with impaired perception, semantic and phonological processing, as well as executive impairments. At the same time, it should be noted that the fMRI data had less statistical significance and the obtained results should be treated with caution.

### 4.8 Limitations and future directions

It should be noted that our work had several limitations. One of them was the selected scale for speech and language assessment. PASS used in our study to measure the severity of aphasia symptoms is not quantitative and allows us to divide the severity of disorders only into five groups. More precise quantification could lead to more precise results and possibly identify more correlations. At the same time, this scale was developed specifically for progressive aphasias evaluation and allows to assess all speech domains necessary for diagnosis and monitoring of patients with PPA. For these reasons and also due to absence of more accurate specialized scales for the Russian-speaking population it was chosen as the best available and suitable option. Other limitations were the relatively small sample size, which was primarily due to the low prevalence of the disease, the predominance of patients with a long disease duration (more than 3 years) and the lack of follow-up data, which did not allow us to accurately separate earlier and later neuroanatomical changes. These limitations must be taken into account when planning further research in this area.

Another relative limitation was choosing ROI-to-ROI analysis with pre-selected areas of interest for functional changes evaluation. Although selected areas covered all main known functionally significant language regions, this method cannot detect possible correlations outside the chosen regions thus artificially limiting the results. To avoid this we conducted a pilot study of whole-brain ROI-to-ROI analysis, which showed involvement of regions outside of the selected areas. However, given the small sample size and low level of statistical significance these results were not discussed in the article. Future works with more participants using seed-to-voxel or whole-brain ROI-to-ROI analysis are needed to obtain these results and expand our findings.

It is also important to note that the data obtained are among the first results for the Russian-speaking population. Thus, some differences between the data obtained and those available in the literature may be due to linguistic differences. This is especially worth considering when evaluating the results of grammar disturbances, as the most variable among different languages. At the same time, there were no native English-speaking patients in our group and we did not conduct a direct comparison between the two groups, which is why it is impossible to draw unambiguous conclusions at this stage. Further research is needed in this area with direct comparison between native speakers of different languages to identify possible differences.

## 5 Conclusion

In this study, we found and analyzed the structural and functional correlates of language impairments in nfvPPA. Our results show that fluency, receptive and expressive agrammatisms and AoS have mainly different functional and structural correlates and thus more likely derive from damage to different brain regions. Functional changes were more widespread, which once again proves that they must be taken into account when studying the disease. In addition to atrophy and FC disruption within the left frontal lobe, we identified the important role of the inferior parietal lobule, especially SMG, in the development of apraxia of speech and receptive agrammatisms, as well as the superior parietal lobule in decreased fluency. Analysis of less frequent nfvPPA symptoms has shown that repetition, naming and single word comprehension impairments may be present in a significant portion of patients with nfvPPA during a long-term course of the disease. Their development is mainly associated with the atrophy propagation to the temporoparietal regions and has a similar pathogenesis to lvPPA and svPPA, sometimes combining both as in case with naming. Repetition impairment, however, may be additionally associated with decreased connectivity between the SMA and the insula.

## Data Availability

The raw data supporting the conclusions of this article will be made available by the authors, without undue reservation.
